# The Jury in Insanity Cases in Texas

**Published:** 1916-10

**Authors:** 


### The Jury in Insanity Cases in Texas.

Among those who attended the convention of the Southern Medical Association was Dr. Thomas W. Salmon, of New York. Dr. Salmon is a neurologist attached to the Rockefeller Institute. He has been in Texas several weeks looking into our methods of caring for the insane, and he will be here several weeks longer engaged in the same work. Then he will formulate his observations and recommendations into a report to the Rockefeller Institute. A copy of it will be given to Governor Ferguson.

Dr. Salmon is quoted as saying that some of the conditions he has found in Texas are "deplorable," and there will be none in Texas to dissent. A severer word than "deplorable" might be used in justness. But Dr. Salmon does not give us quite all the little credit that is due us when he says that "Texas is one of the six States of the Union retaining the jury system of adjudging a person insane." The fact is that we have a law in the statute books which does away with this obsolete method, which, as Dr. Salmon says, obtains now in only six States in the Union. In place of that method, our statute prescribes that a commission of physicians shall be brought together to pass on the question of sanity. But, unhappily a district judge somewhere has discovered that this statute violates the Constitution in a most shameless way, and the higher courts have not had time to tell us whether the district judge is correct or not. Pending the grant of this information, most county judges, we believe, follow the procedure prescribed by the old law. Of course, strictly speaking, Dr. Salmon is correct in saying that we continue to use the obsolete method abandoned by all but six of the States. The only fault to be found with his statement is that it neglects to credit the people and Legislature of the State with their good intention. It is entirely possible, if not somewhat probable, that the higher courts will repair the damage which the district judge did to our new statute.—*Dallas News.*

---

Pigskin Percy, the jockey, was taken ill and a friend gave him the address of a doctor.

Percy came back shortly after and remarked:

"I've got some medicine, but I'm blowed if I went to that doctor of yours."

"Why?" asked his friends, surprised.

"Well," replied Percy, "I was just about to go in when I saw on the door plate, 'Dr. Jones,' and below it 'Ten to one.' When I saw that I said to myself, I'll be hanged if I take any such risks as that, so I went around the corner and saw another plate with the inscription, 'Dr. James," and below, 'Three to five.' The odds were shorter so I went to him.—*Austin American.*

---

"Oh, doctor! I do hope you'll let father smoke again soon. We simply can't get a cent out of him."—*Judge.*

